# An atypical presentation of primary central nervous system lymphoma: A case report

**DOI:** 10.1097/MD.0000000000022062

**Published:** 2020-09-18

**Authors:** Carlen A. Yuen, James Mastrianni, Saad Ali, Peter Pytel, Deric M. Park, Kourosh Rezania

**Affiliations:** a University of Chicago, Department of Neurology, Chicago, IL; b University of Chicago, Department of Radiology, Chicago, IL; c University of Chicago, Department of Pathology, Chicago, IL.

**Keywords:** PNCSL, central nervous system lymphoma, choroid plexus tumor, encephalopathy, limbic encephalitis

## Abstract

**Rationale:**

Primary central nervous system lymphoma **(**PCNSL) involving the choroid plexus is exceedingly rare. The differential diagnosis for choroid plexus enhancing lesions in addition to lymphoma includes infections, sarcoidosis, tuberculosis, papilloma, meningioma, subependymoma, and metastatic lesions.

**Patient concerns:**

A 71-year-old man presented with 3 days of episodic memory loss and gait disturbance. Brain magnetic resonance imaging showed homogenously enhancing lesions with mildly restricted diffusion and T2 hypointensity in the lateral ventricles, as well as T2 hyperintensity and enhancement in the right hippocampus. His episodic memory loss was thought to be secondary to subclinical focal seizures, supported by EEG revealing right temporal lobe epileptiform discharges.

**Diagnoses:**

Large B-cell lymphoma, nongerminal center type was revealed on pathological examination.

**Interventions:**

Stereotactic biopsy of his right thalamic lesion was performed.

**Outcomes:**

The patient underwent induction therapy with high-dose methotrexate, temozolomide, and rituximab, which resulted in complete resolution of the enhancing lesions. He then underwent conditioning chemotherapy with carmustine and thiotepa, followed by autologous stem cell transplantation. His PCNSL remains in remission 42 weeks after the onset of symptoms.

**Lessons:**

We report a patient with multifocal PCNSL involving the choroid plexus, who presented with abnormal gait and episodic confusion and memory loss. PCNSL should be considered in the differential diagnosis of acute encephalopathy among immunocompetent older individuals who have choroid plexus enhancing lesions.

## 1 Introduction

Primary central nervous system lymphoma (PCNSL) is a rare, highly infiltrative central nervous system (CNS) tumor comprising 3% of all newly diagnosed CNS tumors with ∼1900 new cases annually in the United States,^[1]^ with its incidence rising among the patients ≥70 years.^[2,3]^ PCNSL tumors are most commonly CD20+ diffuse large B-cell lymphoma (∼95%), with the T cell variant occurring in approximately 2% of patients.^[1,4]^ PCNSL can occur in the brain parenchyma, cranial nerves, leptomeninges, spinal cord, or intraocular compartment. Choroid plexus PCNSL is an exceedingly rare finding, with only 4 previously reported cases.^[5–8]^

Sixty-five percent of newly diagnosed PCNSL in immunocompetent patients present with solitary lesions and vasogenic edema.^[9]^ Multifocal lesions are more common immunocompromised patients. In patients infected with human immunodeficiency virus (HIV), PCNSL is associated with CD4+ T-cell counts <50 cells/μL and concomitant Epstein–Barr virus (EBV) virus infection.^[10]^ Memory loss and seizures occur more frequently in HIV patients (50% and 38%, respectively) than non-HIV patients (31% and 11%, respectively).^[11]^ PCNSL presents with a varied symptomatology, including acute encephalopathy, which leads to diagnostic evaluation for infectious, inflammatory, metabolic, and neoplastic etiologies. Given that PCNSL is a mimicker of other diseases, PNCSL can become a diagnostic challenge.

## 2 Case report

### 2.1 Patient information

The patient provided a written consent in regards to this publication. We present a 71-year-old Caucasian male pastor with a past medical history of depression, hypertension, and diabetes mellitus who presented to the Emergency Department (ED) for intermittent gait disturbance, episodic confusion, and transient amnesia for recent and past events, including the death of his parents and name of his church. In the ED, he was afebrile (97° F) with no systemic symptoms. His examination was notable for impaired verbal retrieval, visuospatial function, vibratory sensory loss at the toes, and abnormal tandem gait. A noncontrast head computed tomography (CT) showed nonspecific patchy bilateral white matter hypoattenuation, suggestive of microvascular disease.

**Figure 1:**
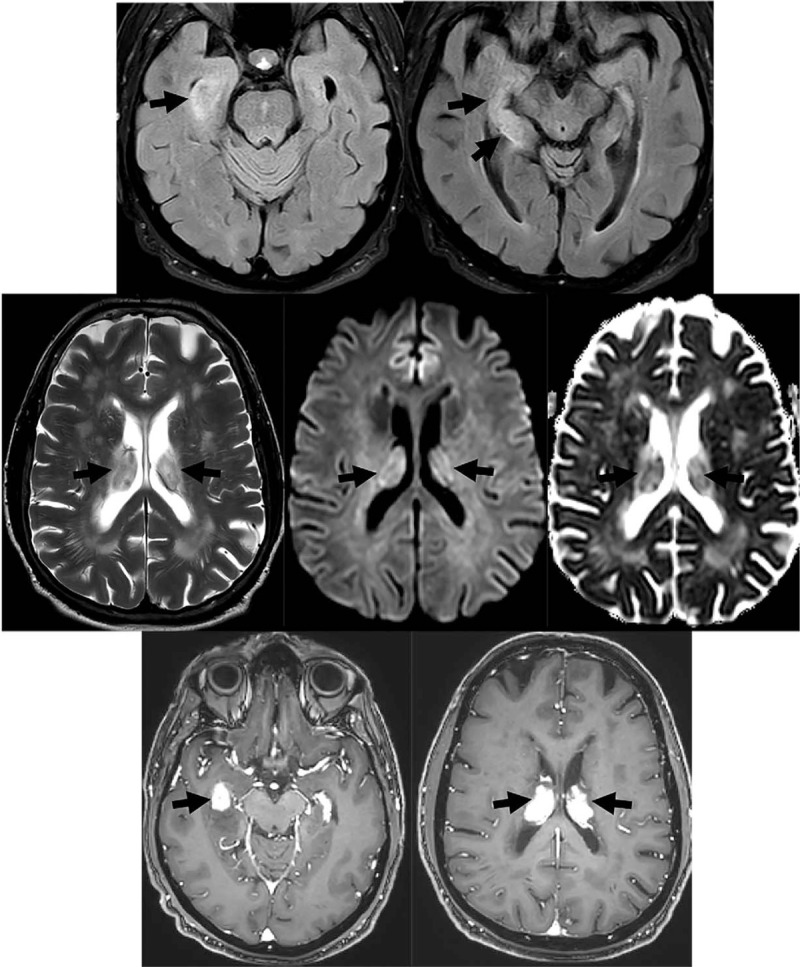
Top row: Axial FLAIR images show increased FLAIR signal in the right medial temporal lobe extending along the right hippocampus, which is mildly enlarged. Middle row: Axial T2, DWI, and ADC map demonstrating additional lesions with mild T2 hypointensity and mild restricted diffusion centered along the inferior aspects of the lateral ventricles bilaterally. Bottom row: Axial post-gadolinium T1-weighted images demonstrating avidly enhancing lesions in the right medial temporal lobe and along the inferior aspects of the lateral ventricles with involvement of the choroid plexus.

Laboratory studies showed a normal complete blood count [white blood cell (WBC) count 8.1 10∗3/μL] and comprehensive chemistry panel. EEG demonstrated right temporal rhythmic delta epileptiform discharges. Brain MRI with and without contrast (Fig. 1) showed diffuse T2 hyperintensity in the right hippocampus with an area of nodular enhancement. Additional homogenously enhancing lesions with mild restricted diffusion and T2 hypointensity were present along the inferior surface of the lateral ventricles extending into the thalami. Lumbar puncture showed opening pressure of 18 cmH_2_0. Cerebrospinal fluid (CSF) analysis showed WBC 48/μL (neutrophils 1%, lymphocytes 88%, monocytes 10%, plasma cells 1%, RBC 97/μL, glucose 64 mg/dL (serum glucose 154 mg/day), and elevated protein of 401 mg/dL. Gram stain and bacterial cultures, and meningitis/encephalitis panel [*Escherichia coli* K1, *Haemophilus influenzae*, *Listeria monocytogenes*, *Neisseria meningitidis*, *Streptococcus agalactiae*, *Streptococcus pneumonia*, cytomegalovirus (CMV), enterovirus, herpes simplex virus 1 (HSV-1), herpes simplex virus 2, human herpes virus 6, Human Parechovirus, Varicella Zoster Virus, Cryptococcus neoformans/Gattii] were negative. CSF cytology was negative, but CSF flow cytometry showed predominantly T cells with an excess of CD8 T cells. There were few B cells, which were polytypic.

Serum serologies for West Nile Virus, Lyme, syphilis, hepatitis B and C, HIV, antinuclear antibody, and antibodies to ds DNA, pANCA, cANCA, and anti MOG IgG1 and serological evaluation for limbic encephalitis, including ANNA-1, ANNA-2, PCA 1, PCA 2, PCA Tr, Amphiphysin Ab, CRMP 5 IgG, Striational Ab, Calcium Channel Ab P/Q Type, Calcium Channel Ab N Type, AChR Ganglionic Ab, and AntiGlial Nuc Ab Type 1, and Volt Gated K Chan Ab were negative. CT scan of chest, abdomen, and pelvis with contrast showed few nonspecific small right lower lobe ground glass opacities, but no definitive evidence of primary malignancy. Serum angiotensin-converting enzyme (ACE) level was normal, but CSF ACE level was elevated (6.8, normal 0.0–2.5 U/L). His episodic confusion and amnesia were thought to be secondary to subclinical focal seizures. He was treated with levetiracetam and intravenous acyclovir.

### 2.2 Surgical and pathological findings

**Figure 2:**
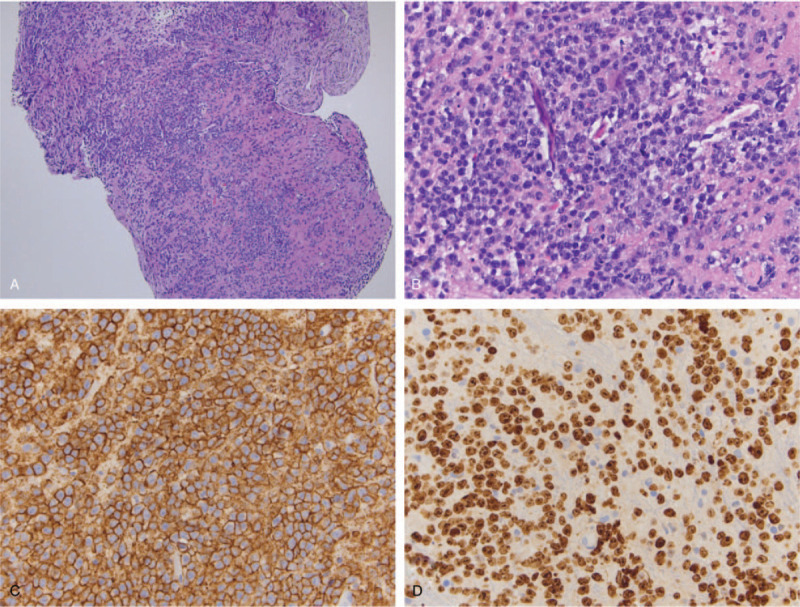
(A) H&E, low power, (B) H&E, high power, (C) Staining for CD20 (B-cell marker), (D) Staining for the proliferative marker MIB-1/Ki-67.

A stereotactic brain biopsy was performed, sampling the right thalamic lesion. Pathological examination revealed cellular infiltrates of large cells with prominent nucleoli. Lesional cells were positive for CD20, MUM1, BCL2, and BCL6, whereas stains for CD3, CD5, CD10, and CD30 were negative, consistent with a diagnosis of large B-cell lymphoma, nongerminal center type (Fig. 2). In situ biopsy hybridization for EBV was negative. Bone marrow biopsy and CT scan of chest, abdomen, and pelvis did not reveal lymphoma infiltration, confirming the diagnosis of PCNSL. Ophthalmologic examination did not show intraocular involvement. Testicular ultrasound showed left epididymal cysts.

### 2.3 Follow-up and outcome

Our patient was treated with 4 cycles of induction therapy of methotrexate, temozolomide, and rituximab (MT-R), which resulted in complete remission of PCNSL. Repeat MRI studies of the brain 15 and 29 weeks after the initial study showed resolution of the enhancing masses in the lateral ventricles and thalami, with residual abnormal FLAIR signal intensity in the right hippocampus. He underwent conditioning chemotherapy with carmustine and thiotepa 38 weeks after the initial hospitalization followed by autologous stem cell transplantation. In his last follow-up 42 weeks after the onset of symptoms, his PCNSL remains in complete remission and his only neurological symptom is short-term memory loss.

## 3 Discussion

Our patient presented with encephalopathy and episodic memory loss resulting from subclinical recurrent complex partial seizures. Abnormal enhancement noted on brain MRI necessitated diagnostic evaluation for viral and nonviral (syphilis, tuberculosis, fungal, mycoplasma) encephalitis, autoimmune (vasculitis, sarcoid, paraneoplastic) disorders, neoplastic infiltration (metastases from systemic carcinomas and lymphomas), and PCNSL. HSV-1 encephalitis accounts for 50% to 75% of identifiable viral etiologies, occurs more commonly in older age, males, and Caucasian race, and is characterized by acute encephalopathy, fever, seizures, and focal neurological deficits.^[12,13]^ CSF pleocytosis is present in most patients, is typically lymphocytic, and may contain a disproportionate number of RBCs. EEG abnormalities include epileptic discharges 89%, periodic lateralized epileptiform discharges 87%, and focal slowing 69%.^[14]^ MRI may reveal temporal lobe hemorrhage, enhancement, and diffusion restriction.^[13]^ HSV-1 could not be immediately excluded in our patient due to his demographics (older age, Caucasian race), presence of temporal lobe abnormality on brain MRI, and focal epileptiform discharges on EEG. Furthermore, fever may be absent in ∼20% to 30% of patients^[12,15]^ and HSV-1 PCR can be negative in the CSF, primarily early in the disease course.^[13,16]^ As a result, our patient was initially treated with IV acyclovir. Consideration of PCNSL in our differential diagnosis precluded use of steroids. Steroid administration can decrease the diagnostic yield by significantly reducing the size of the lesions on biopsy resulting in diagnostic delay.^[17]^

Characteristic radiographic findings in PCNSL are periventricular uniformly contrast-enhancing lesions with restricted diffusion and associated vasogenic edema with mass effect on the lateral ventricles. PCNSL tumors are highly cellular with reduced diffusion of water, leading to diffusion-weighted imaging (DWI) hyperintensity and apparent diffusion coefficient (ADC) hypointensity. This pattern is also found in acute ischemic infarcts and central necrosis in brain abscesses (although without the intense homogeneous enhancement characteristically seen with lymphoma). PCNSL lesions occur most frequently in the cerebral hemispheres (38%), followed by the thalamus/basal ganglia (16%), corpus callosum (14%), intraventricular (12%), and cerebellum (9%).^[9]^ Bilateral ventricular involvement is rare^[3]^ and choroid plexus involvement is exceedingly rare, with only 4 previously reported cases.^[5–8]^ The differential diagnosis for enhancing choroid plexus lesions includes sarcoidosis, tuberculosis, choroid plexus papilloma, meningioma, subependymoma, and metastatic disease.^[8,18,19]^

CSF cytology and stereotactic biopsy are the most frequently used methods to confirm the diagnosis.^[1]^ Cytologic CSF analysis can be falsely negative as seen in our patient and sensitivity can be increased with repeat cytology.^[20]^

CSF ACE levels can be elevated in neurosarcoidosis, demyelinating disease, vasculitis, stroke, and CNS malignant tumors, including astrocytoma, metastatic melanoma, medulloblastoma, and lymphoma,^[21]^ the latter being the case in our patient. Hypoglycorrhachia, also present in our patient, is a finding that can occur due to cellular infiltrates disrupting active glucose transport into the CSF.^[22]^

Further diagnostic evaluation should be performed when PCNSL is suspected, including an ophthalmologic examination for staging with a slit-lamp evaluation to assess for intraocular involvement, which occurs in approximately 20% of PCNSL cases.^[1]^ Contrast-enhanced CT of the chest, abdomen, and pelvis, testicular ultrasound in elderly males, and bone marrow aspirate are also recommended for systemic staging in the diagnostic evaluation for presumptive PCNSL.^[1,23]^ HIV, hepatitis screening, hepatic panel, serum lactate dehydrogenase (LDH), and creatinine clearance should also be performed. Treatment of PCNSL requires intensive chemotherapy of high-dose methotrexate, temozolomide, and rituximab (MT-R).^[1]^

In conclusion, our case demonstrates that homogeneously choroid plexus enhancing lesions should raise suspicion for PCNSL. This case report increases the awareness of the varied clinical presentations of PCNSL and the importance of its consideration in the differential diagnosis of acute encephalopathy.

## Author contributions

**Conceptualization:** Kourosh Rezania.

**Data curation:** Carlen Yuen.

**Supervision:** Kourosh Rezania.

**Writing – original draft:** Carlen Yuen, Kourosh Rezania.

**Writing – review & editing:** James Mastrianni, Saad Ali, Peter Pytel, Deric Park.

## Abbreviations

Abbreviations: MT-R = methotrexate, temozolomide, and rituximab, PCNSL = primary central nervous system lymphoma.
